# Factors Influencing the Early Introduction of Sugar Sweetened Beverages among Infants: Findings from the HSHK Birth Cohort Study

**DOI:** 10.3390/nu12113343

**Published:** 2020-10-30

**Authors:** Vanessa Irvine, James Rufus John, Jane A Scott, Andrew Hayen, Loc Giang Do, Sameer Bhole, Diep Ha, Gregory S. Kolt, Amit Arora

**Affiliations:** 1 School of Health Sciences, Western Sydney University, Penrith, NSW 2751, Australia; 18080546@student.westernsydney.edu.au (V.I.); G.Kolt@westernsydney.edu.au (G.S.K.); 2 Translational Health Research Institute, Western Sydney University, Locked Bag 1797, Penrith, NSW 2751, Australia; james.john@rozettainstitute.com; 3 Rozetta Institute, Sydney, NSW 2000, Australia; 4 School of Public Health, Curtin University, Perth, WA 6845, Australia; jane.scott@curtin.edu.au; 5 Australian Centre for Public and Population Health Research, Faculty of Health, University of Technology Sydney, Ultimo, NSW 2007, Australia; andrew.hayen@uts.edu.au; 6 Australian Research Centre for Population Oral Health, The University of Adelaide, Adelaide, SA 5000, Australia; loc.do@adelaide.edu.au (L.G.D.); diep.ha@adelaide.edu.au (D.H.); 7 Oral Health Services, Sydney Local Health District and Sydney Dental Hospital, NSW Health, Surry Hills, NSW 2010, Australia; Sameer.Bhole@health.nsw.gov.au; 8 Sydney Dental School, Faculty of Medicine and Health, The University of Sydney, Surry Hills, NSW 2010, Australia; 9 Discipline of Child and Adolescent Health, Sydney Medical School, Faculty of Medicine and Health, The University of Sydney, Westmead, NSW 2145, Australia

**Keywords:** sugar sweetened beverages, infants, cohort study, discretionary food, Australia

## Abstract

Understanding the determinants of early introduction of sugar sweetened beverages (SSBs) may assist in designing effective public health interventions to prevent childhood weight related conditions (obesity). This study explores the relationship between family/infant characteristics and the early introduction of SSBs among infants in Sydney, Australia. Mothers (*n* = 934) from an ongoing birth cohort study were interviewed at 8, 17, 34, and 52 weeks postpartum. Multivariable logistic regression analysis was used to identify family/infant factors independently associated with the likelihood of early introduction of SSBs (<52 weeks of age). Of the 934 mothers interviewed, 42.7% (*n* = 399) of infants were introduced to SSBs before 52 weeks. Mothers who were born in Vietnam (adjusted Odds Ratio (AOR) = 2.14; 95% confidence interval (CI) 1.33, 3.47), other Asian countries (AOR = 1.62; 95% CI 1.02, 2.58) as well as single mothers (AOR = 3.72; 95% CI 2.46, 5.62) had higher odds of introducing SSBs early to their infants. Mothers from highly advantaged socioeconomic background (AOR = 0.43; 95% CI 0.28, 0.68), those who breastfed their baby for 17–25 weeks (AOR = 0.60; 95% CI 0.37, 0.99), 26–51 weeks (AOR = 0.65; 95% CI 0.45, 0.94), and 52 weeks or more (AOR = 0.62; 95% CI 0.43, 0.90); and those who introduced solids between 17–25 weeks (AOR = 0.58; 95% CI 0.36, 0.91) and 26 weeks or more (AOR = 0.55; 95% CI 0.34, 0.91) had reduced odds of introducing SSBs early. Tailoring health promotion programs for these vulnerable groups may delay the introduction of SSBs.

## 1. Introduction

Obesity has become increasingly common and is a risk factor for several chronic conditions [1]. In Australia, 8.4% of the total disease burden was attributed to obesity (weight-related conditions) in 2015 [2] with recent findings showing that in 2017–2018 one in four children and adolescents aged 2–17 years were obese or overweight [2,3]. Furthermore, evidence shows that children with obesity are at a higher risk of non-communicable diseases including diabetes, musculoskeletal disorders, dental caries, and depression [4,5]. These effects extend into adolescence and adulthood, and pose significant chronic health concerns [6,7].

Obesity is influenced by several socioeconomic, biological, behavioural and lifestyle factors, making it a complex condition [8]. Factors of interest in the formation of dietary habits and preferences include, but are not limited to, cultural differences, maternal and paternal attitudes, barriers faced by lower income families in acquiring healthy foods, care giver feeding practices such as food being used for rewards or discipline, and levels of care giver knowledge and health literacy [9,10]. Furthermore, studies show that food preferences are established at a young age when children are learning fundamental concepts of what, when, and how much to eat [11,12,13]. These concepts are largely based on the food practices, preferences, attitudes, and cultural beliefs of the caregivers, who therefore, can potentially determine future food choices for their children [12,13]. A child’s early experiences with various tastes and flavours play a significant role in establishing and promoting life-long preferences and behaviours, making this a pivotal area in the development of healthy eating habits [9,14]. This is especially important in the first five years of life as healthy eating habits are fundamental to growth, development and future health trajectories [14,15]. Optimal, healthy neurobehavioral development requires an adequate balance of macro and micronutrients as outlined in the Australian Dietary Guidelines and the Infant Feeding Guidelines [16,17].

The types of food and timing of introduction is very critical, with guidelines reinforcing that discretionary foods should not be introduced before 12 months (52 weeks) as they can have a detrimental effect on long-term health [14,15,16,18,19]. Discretionary foods are defined as energy-dense, but low in nutrients and tend to be high in free sugars, salt, and fat [6,18]. In Australia, for example, discretionary foods, particularly those with a high free sugars content, account for 14% of energy intake for children aged 18 months [6,20,21]. Children even younger at 12–14 months have also been reported to have a high consumption of discretionary foods amounting to 11.2% of their total energy consumption [21]. Sugar sweetened beverages (SSBs), which contribute to a high proportion of free sugars consumed by children, are defined as any liquid that is sweetened with various forms of added sugars such as regular soda, fruit drinks, sports and energy drinks, sweetened waters, and coffee/tea with added sugar [17].

It has been reported that Australian 2-year-old children consume on average 22.5 g/day of free sugars [22]. This exceeds the Australian Dietary Guidelines recommended intake of no more than 19 g of free sugars daily (approximately five sugar cubes) estimated for even older children aged 4–6 years [17]. The World Health Organization recommendation of limiting consumption of free sugars to less than 10% of total energy intake (ideally less than 5%) is also contradicted by these current statistics [18,23]. As such, it is paramount to establish how and where children are exposed to these levels of sugar consumption. The literature shows that 60% of all SSBs were consumed within the home environment, indicating that family and parental food purchasing decisions are significant drivers of SSB consumption by children [24]. In the case of those of even younger age such as infants, their ability to choose their diet is even more limited with strong evidence showing that diets of children of pre-school age or younger depend almost solely on their parents or carers [25]. This may make them vulnerable to unhealthy parental feeding habits [25].

The Australian Dietary Guidelines and Australian Infant Feeding Guidelines state that discretionary foods should not be given to infants under 12 months (52 weeks) of age [16,17]. Additionally, the guideline also states that fruit juices or other sweetened beverages as well as the addition of sweeteners including honey are not recommended for infants and should be avoided [16]. Despite the significance of such information, a knowledge gap exists as to how accurately the dietary guidelines are being followed, and what factors influence parents and primary caregivers to adhere to, or even be aware of, the recommendations. Several factors related to the family and infant have been identified in the literature as influential in the early introduction of SSBs. However, these factors differ by country, geographical region, and ethnic groups. In countries such as the UK and the US, breastfeeding, socioeconomic status, and education level are known predictors of SSB introduction [26,27]. There is, however, limited information from Australia exploring these factors in an ethnically diverse population such as in South Western Sydney. As Australia is a multicultural country, focusing on ethnic minorities is crucial, and identifying the factors that put families at risk of poor eating habits is an important step in developing targeted health promotion programs that may increase the rate of adherence to the dietary guidelines. This could be a significant action towards reducing further health concerns associated with poor infant diet, particularly high-sugar diets. Therefore, this study aims to investigate the family and infant characteristics associated with the early introduction of sugar sweetened beverages.

## 2. Materials and Methods

### 2.1. Study Population

This study is a secondary analysis of data from the ongoing, longitudinal “Healthy Smiles Healthy Kids (HSHK)” birth cohort study that recruited mother-infant dyads in South Western Sydney at the time of the child’s birth (the complete details of the protocol of this birth cohort study have been published [28]). Women who gave birth to live infants with no known health conditions in seven public hospitals located in the former Sydney South West Area Health Service (now classified as Sydney and South Western Sydney Local Health Districts) were approached to participate in 2010. Eligible mother-infant dyads were recruited at the first home post-natal visits at 4–6 weeks by Child and Family Health nurses. Written consent was obtained at this time and interpreter services for non-English speakers were provided along with written material in the respective languages of the women [28]. This study was conducted in public hospitals in order to better recruit the under-represented population groups found in Sydney including socioeconomically disadvantaged and ethnically diverse groups [29].

### 2.2. Data Collection

Initially, a baseline telephone interview (at 8 weeks postpartum) was conducted to gather demographic and infant information. This information included feeding practices such as duration and exclusivity of breastfeeding, age of introduction of complementary foods including discretionary foods, and family demographic information such as maternal marital status, socioeconomic status, and parental country of birth. Follow-up telephone interviews were conducted at 17, 34, and 52 weeks postpartum. At every interview, the information collected included infant feeding practices such as breastfeeding, the use of infant formula, and core and discretionary foods. The core foods were comprised of the five food groups: grain foods, fruit, vegetables, lean meat/poultry/fish/eggs, and dairy products. Water was also included as a core food. Discretionary foods were categorised into two main groups: foods high in saturated fats and foods/drinks with added sugars. SSBs, which are the focus of this paper, fall into the last category of discretionary foods. The structured questionnaires were adapted from the first and second Perth Infant Feeding studies [30,31], Iowa Fluoride Study [32], and the NSW Child Health Questionnaire [33] to determine if SSBs had been introduced and to identify the factors associated with early introduction. The pilot data are published elsewhere [34].

### 2.3. Outcome Measure

At each phase of the interviews, mothers were asked the question: How often was your baby fed “X”, from a list of foods and beverages, in the last seven days, and the age (in weeks) at which they first tried them. The earliest age (in weeks) that the infant had first tried each item was recorded. If the child was not introduced to any complementary food by the baseline period (8 weeks), then the responses were taken from the follow-up interviews at 17, 34 weeks and 52 weeks. This study specifically focused on the introduction of flavoured milk, fruit juice, soft drinks, cordial, sports drinks, iced tea/coffee, powdered drinks, and flavoured mineral water. Early introduction of SSBs was considered to be before 52 weeks of age [13,16,17,26,35].

### 2.4. Factors Influencing the Early Introduction of SSBs

A number of family and infant characteristics were recorded. Family characteristics included maternal age (in years), mother’s level of education (<year 12, i.e., final year of high school), year 12 completed, college/TAFE, University), mother’s occupation (home duties, managerial/professional, sales/clerical, unskilled), marital status (with a partner, single), mother’s and the partner’s country of birth (Australia, China, Vietnam, other Asian countries, Middle East/Africa, Other), parity (1, 2+), and socio-economic status (SES), which was defined using the residential postcode as per the Index of Relative Socioeconomic Advantage and Disadvantage (IRSAD) [36].

Infant characteristics included infant’s sex (male, female), and age at which breastfeeding was ceased (<17 weeks, 17–25 weeks, 26–51 weeks, ≥52 weeks), and solid foods were introduced (less than 17 weeks, 17–25 weeks, 26 or more weeks).

### 2.5. Statistical Analysis

Data were entered and analysed using the Statistical Package for Social Sciences (SPSS) Version 25 (SPSS for MacOS, SPSS Inc., Chicago, IL, USA). Descriptive analyses were performed to describe the proportion of infants introduced to SSBs before 52 weeks. Continuous variables were expressed as mean and standard deviation (SD) whereas frequency counts of categorical variables were shown as percentages. This was followed by univariable logistic regression analysis to determine the factors associated with early introduction of SSBs. Multivariable regression models were used to estimate the association between the exposure variables (family and infant co-variates), and the outcome (early introduction of SSBs) to estimate adjusted odds ratio (AOR) and associated 95% confidence intervals (95% CI). Co-variates were entered into the explanatory model based on evidence from previously published studies. The full model was reduced using backward stepwise regression to remove non-significant variables. Dropping non-significant variables that could potentially affect the model fitness was avoided by assessing the fitness of model at every step of the analyses. Only variables that were statistically associated (*p* < 0.05) with the outcome measure remained in the final model. All variables in the final model were variables from which, when excluded, the change in log likelihood ratio compared with the corresponding chi-squared (χ^2^) test statistic on the relevant degrees of freedom was significant.

### 2.6. Ethics Approval and Consent to Participate

Ethics approval for this study were provided by the former Sydney South West Area Health Service—RPAH Zone (ID number X08-0115), Liverpool Hospital, University of Sydney, and Western Sydney University. All participants agreed to participate in this study by signing a written consent form.

## 3. Results

### 3.1. Response Rate and Baseline Characteristics

**Figure 1:**
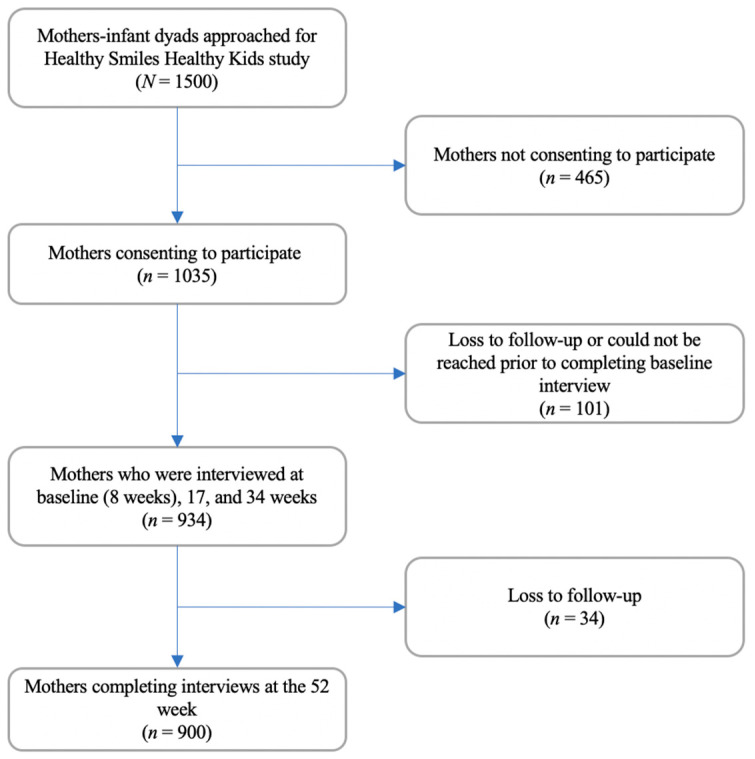
Flowchart of study sample recruitment and retention.

Of the 1500 mothers approached, 1035 mothers consented to participate (69.0% response rate) in the HSHK study. Mothers who declined to participate (*n* = 465) were not statistically different from participating mothers in terms of age (χ^2^ = 4.75, *p* = 0.153), educational level (χ^2^ = 6.65, *p* = 0.328) and infant feeding method (χ^2^ = 2.46, *p* = 0.813). A total of 934 mother-infant dyads completed the interviews at 8, 17, and 34 weeks postpartum, and 900 dyads completed the interview at 52 weeks (Figure 1). There were no statistically significant differences with respect to maternal age, education level, and infant feeding method of those who completed the 52 weeks interview and those who withdrew from the study (data not reported).

**Figure 2:**
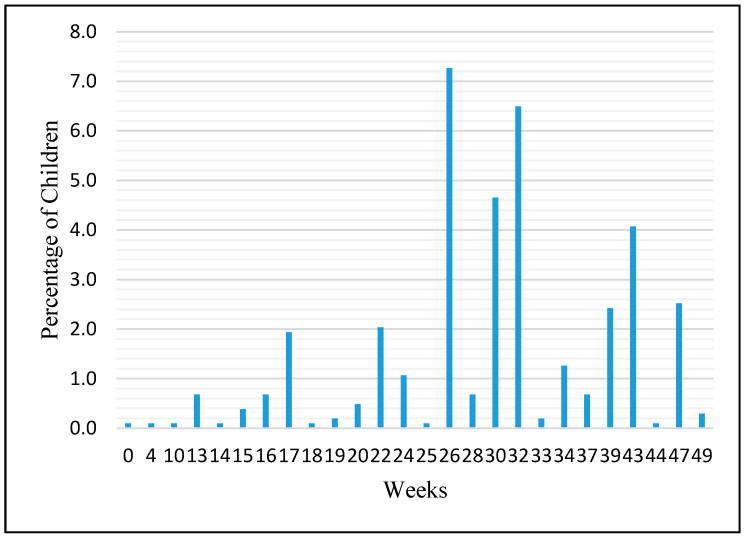
Introduction of sugar sweetened beverage (SSB) by infant age in weeks.

Family and infant characteristics of the study population are shown in Table 1. Of the 934 participating mothers, 399 reported introducing SSBs to the infant diet before 52 weeks of age (42.7%). The median age for the introduction of SSBs was 30 weeks (interquartile 1: 26 and interquartile 3: 37) with the peak times of introduction at 26 and 37 weeks (Figure 2).

### 3.2. Univariable Logistic Regression

The univariable analysis (Table 2) indicated that the introduction of SSBs was associated with various family and infant characteristics. The odds of introducing SSBs before 52 weeks of age decreased with increasing maternal age (odds ratio (OR) = 0.96; 95% CI = 0.93, 0.98; *p* < 0.00). Similarly, mothers who had completed a college (OR = 0.48; 95% CI = 0.32, 0.75; *p* < 0.00) or university (OR = 0.38; 95% CI = 0.54, 1.24; *p* < 0.34) level of education, were living in least disadvantaged postcodes (OR = 0.32; 95% CI = 0.22, 0.47; *p* < 0.00) and had occupations categorised as managerial/professional (OR = 0.44; 95% CI = 0.30, 0.65; *p* < 0.00) had reduced odds of introducing SSBs to their infant’s diet before 52 weeks of age. Vietnamese-born mothers (OR = 2.65; 95% CI = 1.78, 3.95; *p* < 0.00) were more likely to introduce SSBs early to their infant’s diet. Similarly, single mothers (OR = 3.85; 95% CI = 2.66, 5.59; *p* < 0.00) were almost four times more likely to introduce SSBs early than mothers who had a partner. Of the infant characteristics, those who had solid food introduced between 17–25 weeks (OR = 0.46; 95% CI = 0.30, 0.70; *p* < 0.00) and those who continued breastfeeding for longer had reduced odds of introducing SSBs early (OR = 0.47; 95% CI = 0.30, 0.73; *p* < 0.00).

### 3.3. Multivariable Logistic Regression

The multivariable analysis (Table 2) showed single mothers had almost four times higher odds of introducing SSBs before 52 weeks (AOR = 3.72, 95% CI = 2.46, 5.63; *p* < 0.00) than women with a partner. Compared to mothers who were born in Australia, those born in Vietnam had two times higher odds (AOR = 2.14; 95% CI 1.32, 3.47; *p* < 0.00) and mothers born in other Asian countries such as India had 62% higher odds (AOR = 1.62; 95% CI 1.02, 2.58; *p* < 0.04) of introducing SSBs earlier than 52 weeks. Additionally, mothers living in the most advantaged areas (deciles 9 and 10) had 57% lower odds (AOR = 0.43, 95% CI = 0.28, 0.68; *p* < 0.00) of introducing SSBs before 52 weeks compared with those living in the most socially disadvantaged areas. Mothers who introduced solid foods into the infant’s diet between 17–25 weeks had 42% lower odds (AOR = 0.58; 95% CI 0.36, 0.91; *p* < 0.01) of introducing SSBs before 52 weeks compared with mothers who introduced solid foods earlier than 17 weeks. Introducing solid foods at 26 weeks or later had 45% lower odds (AOR = 0.55; 95% CI 0.34, 0.91; *p* < 0.02) of introducing SSBs earlier than 52 weeks. Lastly, mothers who breastfed for 17–25 weeks (AOR = 0.60; 95% CI 0.37, 0.99; *p* < 0.04), 26–51 weeks (AOR = 0.65; 95% CI 0.45, 0.94; *p* < 0.02), and 52 weeks or longer (AOR = 0.62; 95% CI 0.43, 0.90; *p* < 0.01) were 40%, 35%, and 38% less likely to introduce SSBs early, respectively.

## 4. Discussion

This study explored family and infant characteristics associated with the introduction of SSBs to the diet of infants under 52 weeks of age in Sydney, Australia. Findings showed that of the 934 mothers, 399 (42.7%) had introduced SSBs to their infant before 52 weeks of age and the main predictors that were significant were maternal marital status, maternal country of birth, socioeconomic status (as determined by postcode and IRSAD), breastfeeding duration and time of introduction of complementary food. Although the Australian Dietary Guidelines recommends that no SSBs be introduced before 52 weeks and that added sugars should be avoided, it is evident that these guidelines were not met by 42.7% of the study population [16,17]. Given the literature regarding the importance of early childhood experiences in developing dietary and taste preferences, this is an important finding [11,12]. This finding is consistent with studies that indicate that 47% of older children reported consumption of SSBs on the day they were surveyed and 2018 data reporting that 48% of adults consumed sugar sweetened beverages at least once per week [37,38,39]. The 2017–2018 National Health Survey in Australia also found that 44.8% of children aged 2 to 17 years consumed SSBs at least once a week [38]. There is, however, limited data specifically for SSB introduction to infants under 52 weeks of age in Australia as the National Infant Feeding survey focuses mainly on breastfeeding rates [39]. This issue is not unique to Australia with data published from the National Health and Nutrition Examination Survey in the United States showing that 38.7% of children aged 6–11 months consumed 100% fruit juice and 5% were introduced to other SSBs [40]. Additionally, another US study showed that 25% of infants aged 6–8 months already regularly consumed 100% fruit juice [41].

The present study identified that single mothers were more likely to introduce SSBs to their infant than partnered mothers. This is a common finding in previous studies with a number of them indicating that parents’ being married, or cohabiting was associated with lower SSB consumption [42,43]. Similarly, children of single-parent households have been shown to have reduced levels of physical activity as well as diets high in fat and sugar and less fresh vegetables and fruits than children from dual-parent households, increasing their risk of developing conditions such as obesity [44]. It has been suggested that single parent families experience financial strain and may contribute to food insecurity given the likelihood that single parent households have a lower total income [45]. Additional factors such as reduced time for domestic tasks such as grocery shopping and cooking arguably lead to a higher reliance on processed convenience foods as well as less expensive and less healthy options [45,46,47].

Socioeconomic status has been shown to be a significant predictor of SSB consumption with the data demonstrating the general trend of mothers living in disadvantaged areas being more likely to introduce SSBs than mothers living in less disadvantaged areas [13,35]. It has been suggested that as SSBs are relatively low in price when compared to healthier alternatives, they are associated with higher consumption by families of lower socioeconomic status. It has been reported that on average, higher socioeconomic populations have a more favourable nutrient intake overall [48], with the National Nutrition and Physical Activity survey (2011–2012) reporting that people living in areas with the highest levels of socioeconomic disadvantage were more likely to drink sweetened beverages [6,48]. Socioeconomic disadvantage is frequently linked with lower consumption of fruits and vegetables and higher consumption of energy-dense foods [46], as attributes of the built environment, such as grocery options and advertising can play a significant role in disparities in diet [49]. It is likely that living in a disadvantaged neighbourhood, which characteristically have a high density of fast food outlets and convenience stores selling heavily processed and energy-dense foods, can limit accessibility to affordable healthy food options [49]. This is an important finding as it has important and far-reaching implications for health inequities in the population. While other literature has identified a link between parental employment status and early consumption of high-sugar foods and drinks [50], this study has not found that association.

Maternal country of birth was shown to be a significant factor in the early introduction of SSBs into infants’ diets with Vietnamese-born mothers and mothers born in other Asian countries, such as India, Cambodia, and Nepal, having higher odds of introducing SSBs than Australian-born mothers. Consistent with the study findings, other studies have reported that Indian-born mothers were more likely to introduce their child to foods and drinks with free sugars than mothers from other countries and findings from Cambodia and Nepal showed that 32% of children in Phnom Penh and 16.2% of children in Kathmandu valley had consumed SSBs within the first year of life [13,51]. Beyond this, there are, unfortunately, very few studies available for direct comparison that specifically examine maternal country of birth and infant SSB consumption. The findings that the consumption of SSBs is more common in children of ethnic minorities is concerning as targeted marketing to ethnic minorities is common in some countries such as the United States [52]. The finding that the consumption of SSBs is more common in children of ethnic minorities may point to a lack of culturally appropriate and linguistically diverse post-natal care and education. It is possible that there is a lack of understanding surrounding the sugar content and recommendations regarding some SSBs such as fruit juice and water-based drinks when compared with other more obvious discretionary foods such as carbonated SSBs. Additionally, this could indicate a lack of access to advice from extended family for Vietnamese and other Asian country-born mothers in Western Sydney represented in the present study which could account for the reduction in SSB introduction when compared with studies from other areas of Australia. This could be linked to several other factors including the amount of time the mother had lived in Australia, available support and advice from culturally appropriate sources, and support from healthcare professionals. However, there is a large data gap in this area which requires further research.

Mothers who introduced solid foods later than 17 weeks were also shown to be less likely to introduce SSBs into their infant’s diet by 52 weeks. Introducing complementary foods at 26 weeks or later had an even less likelihood. This association has been reported in an earlier Australian study that showed that children who received complementary foods before 17 weeks were almost twice more likely to have been introduced to SSB by 52 weeks than those who received complementary foods at or after 17 weeks of age [14]. It is likely that this association reflects a general lack of awareness of current infant feeding recommendations both in terms of when to begin complementary feeding and what foods are suitable during this transition period to the family diet. The Australian Infant Feeding Guidelines are written for health care professionals and not for caregivers per se. They contain relatively little information on the specific types and amounts of foods that should be eaten by children and there is a call for stronger recommendations so that parents understand the specific foods children should and should not be eating [53,54].

Lastly, breastfeeding duration proved to be a significant factor in the prediction of early SSB introduction. The current study showed that mothers who breastfed for longer than 17 weeks all had a decreased likelihood of introducing SSBs to their infants’ diets within the first year of life. This supports the findings of earlier studies that reported that breastfeeding for less than 26 weeks was associated with introduction of high sugar foods [13]. It is likely that women who breastfed beyond 17 weeks are generally more familiar with the current infant feeding recommendations or it is possible that mothers who are able to breastfeed for longer are in a situation where they are able to make healthier choices for their infants. It may be in the form of longer maternity leave, which is associated with higher paying jobs, partner financial support, or family assistance; all of which are linked with better health outcomes. Further interventions targeting mothers who are unable to breastfeed for longer than the recommended amount of time should include information regarding alternate beverages.

It is also important to address the potential misconceptions regarding SSBs as it is possible that many parents/caregivers do not consider that fruit juice falls into the SSB category. A study comparing traditional SSBs such as soda and 100% orange juice identified a similar sugar energy density profile and dose-response association of 100% fruit juice to cardiometabolic disease [55]. It is important to note that failing to address public misconceptions may result in continued misinterpretation of nutritional information and may be a key reason why guidelines are not being followed.

### 4.1. Strengths and Limitations

This study provides valuable insight into the current level of compliance with Australian guidelines regarding infant feeding practices within the first year of life and the introduction of SSBs during this time. This study also highlights mothers from socioeconomically disadvantaged and culturally diverse groups, who are often under-represented in literature of this kind. There are a limited number of studies that have investigated the factors associated with the early introduction of SSBs, which makes the data obtained in this study important for future research and the implementation of improved policies and practice. It is critical that the vulnerable groups highlighted in this study are considered so that informed and effective programs can be applied. The study had a good response rate of 69%, which was acceptable for the validity of this study. Infant feeding data were collected longitudinally at regular intervals in the first year of life, which minimised recall bias. As South Western Sydney is a culturally and linguistically diverse population, interpreters were used to ensure clear understanding of survey and responses that accurately represent the population. The findings may assist in the development of future health promotion policies and strategies in multiple populations of women to increase the quality of infant diets and contribute to better long-term health outcomes.

There are, however, several limitations of this study with the major limitation being that while SSB introduction was recorded, the amount and frequency of consumption were not measured. Therefore, we do not know if individual children were frequent or infrequent consumers of SSB. This is an important distinction as frequency may impact the long-term health outcomes. Additionally, as the participants were recruited in public hospitals, the associations drawn in the study may not reflect the associations at the wider population level in New South Wales. The design of the study also introduces the possibility of social desirability bias as the data were self-reported by the subjects and knowing that the study had an oral health focus, some participants may have misreported if and when they first gave their infants SSBs. Lastly, for various explanatory variables such as country of birth, the number of women in the category were small, thus causing a rare event bias that was reflected as a large confidence interval around the odds ratio. Further studies with a larger sample of women may be required to provide more statistically robust findings in those areas.

### 4.2. Policy Implications

High sugar intake has been linked to obesity and related non-communicable diseases, for which the healthcare costs in Australia are estimated to be more than $6.57 billion per year with additional indirect costs in lost productivity and tax revenue [6,56]. Additionally, early consumption of SSBs has been linked to the development of dental caries at early school age making it a major challenge to future oral health [57,58]. SSBs provide a high dose of free sugars with little other nutritional value and as such, should be a clear focus for public health action [17,18]. Several approaches for reducing sugar consumption in Australia have been proposed with the implementation of a sugar tax being one of the most extensively discussed methods [56]. As it has been shown that SSBs are largely consumed due to their lower price-tag and ready availability, a tax may lead to a reduction in purchase and consumption [24]. It might be important for the vulnerable groups in this present study as the SSB tax has seen a reduction in SSB consumption across the board but especially in low-income households [59]. This has also been trialed in Europe with varying degrees of success, but it has resulted in a general downward trend in SSB consumption [56].

At a national policy level, food supply and agricultural policies have a strong impact on public health and population diet [60]. Government support for certain crops influence the marketable prices and availability for consumers [61]. Particularly in the US, government policy has made sugars and fats some of the most inexpensive crops to produce and this indirectly influences food processors to include fats and sugars in their produce [60]. Given the evidence for the negative effects of sugars and fats on the population, influencing agricultural policies to support healthier crops may make healthier food alternatives more accessible. A US study showed that a 10% reduction in price for fruits and vegetables increases consumption by 7.2%, which indicates that reducing the price may increase the intake of healthy foods [62].

## 5. Conclusions

This study reports on the factors associated with early introduction of SSBs among women and infants in South Western Sydney, Australia. It highlights the important socio-demographic determinants that influence SSB introduction in infants among mothers in South Western Sydney. As one of the most culturally diverse and disadvantaged populations in Australia, special considerations in the form of educational awareness and culturally appropriate interventions are needed to meet the needs of this population. This study suggests a need for better compliance with infant feeding guidelines regarding SSB introduction. It may be important to target the areas of strong predictors for this behaviour such as mothers with high disadvantage, single mothers, both Australian-born and mothers of other ethnicities, and mothers who introduce complementary foods earlier than 17 weeks or breastfeed for less than 17 weeks. Future development of health promotion policies and strategies should take these factors into account and develop accessible and culturally appropriate strategies to ensure better population health outcomes.

## Figures and Tables

**Table 1:** Family and infant characteristics associated with early introduction of SSBs (*n* = 934).

Characteristic	N ^a^ (%)	SSB Introduction before 52 Weeks	
		Yes (*n* = 399)	No (*n* = 633)
Family Characteristics			
Maternal age in years, mean (SD) *	31.23 (5.33)	30.51 (5.8)	31.77 (4.9)
Marital status of mother			
With a partner	779 (83.4)	291(37.4)	488 (62.6)
Single	155 (16.6)	108 (69.7)	47 (30.3)
Mother’s country of birth			
Australia	437 (46.8)	165 (37.8)	272 (62.2)
China	57 (6.1)	15 (26.3)	42 (73.7)
Vietnam	133 (14.2)	82 (61.7)	51(38.3)
Other Asian country	109 (11.7)	48 (44.0)	61(56.0)
Middle East/Africa	81 (8.7)	35 (43.2)	46 (56.8)
Other	117 (12.5)	54 (46.2)	63 (53.8)
Maternal Education			
<12	168 (18)	96 (57.1)	72 (42.9)
Year 12 completed	192 (20.6)	100 (52.1)	92 (47.9)
College/TAFE	170 (18.2)	67 (39.4)	103 (60.6)
University	404 (43.3)	136 (33.7)	268 (66.3)
Mother’s Occupation			
Home duties	169 (18.1)	88 (52.1)	81 (47.9)
Managerial/professional	301 (32.2)	98 (32.6)	203 (67.4)
Sales/Clerical	296 (31.7)	129 (43.6)	167 (56.4)
Unskilled	168 (18)	84 (50.0)	84 (50.0)
Index for Relative Socioeconomic disadvantage			
Deciles 1 and 2	303 (32.4)	159 (52.5)	144 (47.5)
Deciles 3 and 4	220 (23.6)	111 (50.5)	109 (49.5)
Deciles 5 and 6	30 (3.2)	9 (30.0)	21 (70.0)
Deciles 7 and 8	160 (17.1)	62 (38.8)	98 (61.2)
Deciles 9 and 10	221 (23.7)	58 (26.2)	163 (73.8)
Number of children			
1	465 (49.8)	199 (42.8)	266 (57.2)
2+	469 (50.2)	200 (42.6)	269 (57.4)
Infant Characteristics			
Infant sex			
Male	477 (51.1)	198 (41.5)	279 (58.5)
Female	457 (48.9)	201 (44.0)	256 (56.0)
Introduction of solid foods			
Less than 17 weeks	111 (12.2)	67 (60.4)	44 (39.6)
17–25 week	499 (54.7)	205 (41.1)	294 (58.9)
≥26 weeks	303 (33.2)	126 (41.6)	177 (58.4)
Breastfeeding duration			
Less than 17 weeks	358 (38.4)	192 (53.6)	166 (46.4)
17–25 weeks	108 (11.6)	38 (35.2)	70 (64.8)
26–51 weeks	228 (24.5)	83 (36.4)	145 (63.6)
≥52 weeks	238 (25.5)	86 (36.1)	152 (63.9)

^a^ The total of the categories do not always add up to 934 due to missing or incomplete data for some items. * Data presented as N (%) unless specified otherwise, maternal age is continuous therefore percentages not reported. SD- Standard Deviation

**Table 2:** Unadjusted and adjusted odds ratio for early introduction of SSBs (<52 weeks).

Variable	N ^a^	Unadjusted Odds Ratio				Adjusted Odds Ratio			
		Odds Ratio	95% CI	*p* Value	Overall *p* Value	Odds Ratio	95% CI	*p* Value	Overall *p* Value
Maternal age (years)	934	0.96	0.93–0.98	0.00			Not retained in final model		
Marital status of mother					0.00				
With a partner	779	1.00	(Reference) ^b^			1.00	(Reference)		
Single	155	3.85	2.66–5.59	0.00		3.72	2.46–5.62	0.00	0.00
Maternal Education						Not retained in final model			
Below year 12	168	1.00	(Reference)		0.00				
Year 12 completed	192	0.81	0.54–1.24	0.34					
College/TAFE	170	0.48	0.32–0.75	0.00					
University	404	0.38	0.26–0.55	0.00					
Mother’s Occupation						Not retained in final model			
Home duties	169	1.00	(Reference)		0.00				
Managerial/professional	301	0.44	0.30–0.65	0.00					
Sales/Clerical	296	0.71	0.49–1.04	0.07					
Unskilled	168	0.92	0.60–1.41	0.70					
Mother’s country of birth					0.00				0.01
Australia	437	1.00	(Reference)			1.00	(Reference)		
China	57	0.58	0.32–1.09	0.09		0.71	0.37–1.37	0.31	
Vietnam	133	2.65	1.78–3.95	0.00		2.14	1.32–3.47	0.00	
Other Asian country	109	1.29	0.85–1.98	0.23		1.62	1.02–2.58	0.04	
Middle East/Africa	81	1.25	0.78–2.03	0.35		1.18	0.68–2.04	0.56	
Other	117	1.41	0.94–2.13	0.10		1.39	0.89–2.18	0.15	
Index for Relative Socioeconomic disadvantage					0.00				0.00
Decile 1 and 2	303	1.00	(Reference)			1.00	(Reference)		
Decile 3 and 4	220	0.92	0.65–1.30	0.65		1.18	0.80–1.74	0.42	
Decile 5 and 6	30	0.38	0.17–0.87	0.02		0.46	0.19–1.11	0.08	
Decile 7 and 8	160	0.57	0.39–0.85	0.05		0.75	0.48–1.19	0.23	
Decile 9 and 10	221	0.32	0.22–0.47	0.00		0.43	0.28–0.68	0.00	
Infant sex					0.00	Not retained in final model			
Male	477		(Reference)						
Female	457	0.44	0.85–1.43	0.44					
Number of Children						Not retained in final model			
1	465	1.00	(Reference)		0.247				
2+	469	1.07	0.827–1.384	0.606					
									
Introduction of solid foods					0.00				0.04
Less than 17 weeks	111	1.00	(Reference)			1.00	(Reference)		
17–25 weeks	499	0.46	0.30–0.70	0.00		0.58	0.36–0.91	0.01	
≥26 weeks	303	0.47	0.30–0.73	0.00		0.55	0.34–0.91	0.02	
Breastfeeding duration					0.00				0.03
Less than 17 weeks	358	1.00	(Reference)			1.00	(Reference)		
17–25 weeks	108	0.47	0.30–0.73	0.00		0.60	0.37–0.99	0.04	
26–51 weeks	228	0.50	0.35–0.70	0.00		0.65	0.45–0.94	0.02	
≥52 weeks	238	0.49	0.35–0.68	0.00		0.62	0.43–0.90	0.01	

^a^ The total of the categories do not always add up to 934 due to missing or incomplete data for some items. ^b^ The first level of each variable was used as the reference category in the statistical analysis as based on the literature. CI—Confidence Interval.
